# Technical efficiency of selected hospitals in Eastern Ethiopia

**DOI:** 10.1186/s13561-017-0161-7

**Published:** 2017-06-20

**Authors:** Murad Ali, Megersa Debela, Tewfik Bamud

**Affiliations:** 1 Department of Economics, Haramaya University, College of Business and Economics, Dire Dawa, Ethiopia; 2 Department of Economics, Hawasa University, College of Business and Economics, Awasa, Ethiopia

**Keywords:** Technical inefficiency, DEA, Scale efficiency, Hospitals, Malmquist total factor productivity

## Abstract

This study examines the relative technical efficiency of 12 hospitals in Eastern Ethiopia. Using six-year-round panel data for the period between 2007/08 and 2012/13, this study examines the technical efficiency, total factor productivity, and determinants of the technical inefficiency of hospitals. Data envelopment analysis (DEA) and DEA- based Malmquist productivity index used to estimate relative technical efficiency, scale efficiency, and total factor productivity index of hospitals. Tobit model used to examine the determinants of the technical inefficiency of hospitals. The DEA Variable Returns to Scale (VRS) estimate indicated that 6 (50%), 5 (42%), 3 (25%), 3 (25%), 4 (33%), and 3 (25%) of the hospitals were technically inefficient while 9 (75%), 9 (75%), 7 (58%), 7 (58%), 7 (58%) and 8 (67%) of hospitals were scale inefficient between 2007/08 and 2012/13, respectively. On average, Malmquist Total Factor Productivity (MTFP) of the hospitals decreased by 3.6% over the panel period. The Tobit model shows that teaching hospital is less efficiency than other hospitals. The Tobit regression model further shows that medical doctor to total staff ratio, the proportion of outpatient visit to inpatient days, and the proportion of inpatients treated per medical doctor were negatively related with technical inefficiency of hospitals. Hence, policy interventions that help utilize excess capacity of hospitals, increase doctor to other staff ratio, and standardize number of inpatients treated per doctor would contribute to the improvement of the technical efficiency of hospitals.

## Background

The health care system of many countries in Sub-Saharan Africa including Ethiopia faces resource constraints to provide quality health services to the people. The shortage of health care resources may be related to poor economic performance, rapid population growth, and a decline in public spending. Moreover, in Sub-Saharan Africa, communicable, maternal, nutritional, and new borne diseases continue to dominate and putting stress on the already scarce health care resources of these countries [1].

Hospitals consume a larger proportion of the total public health budget. Even though the percentage vary from country to country, hospitals in Sub-Saharan African countries consume a larger proportion of public health care resources. The situation in Ethiopia is like other Sub-Saharan countries. Hence, the efficiency of hospitals need to be given due attention as the budget they consume is enormous.

It is recognized that improved efficiency is one of the main goals of health systems [2]. Health policy makers in Africa have also stressed the need to utilize the scarce health sector resources efficiently [3]. A growing number of countries in Africa have undertaken health facility efficiency study to guide the development of interventions to reduce wastage of scarce health system resources.

Data Envelopment Analysis (DEA) has been used to analyze technical efficiency of hospitals in several Sub-Saharan African countries. Different studies have used different inputs and outputs to measure efficiency. For instance, studies conducted in Eritrea [4], Botswana [5], Benin [6] and Burkina Faso [7] are among the few. Most of the studies focused on measuring the first stage of efficiency analysis. Few studies conducted the second stage analysis to examine the determinants of technical (in) efficiency using panel data. This study uses panel data for six-year-round (that is between 2007/08 to 2012/13) data for each hospital. Panel data enables analysis hospitals productivity changes. The benefit of having multiple observations (panel data) on the same units allows controlling for unobserved heterogeneous characteristics of hospitals. And thus, facilitate causal inference [8]. The second stage analysis involves converting the DEA efficiency score into inefficiency score and running regression against some factors. In this regard, [9] analyzed technical efficiency and productivity in South Africa using panel data.

In the case of Ethiopia, few studies have been conducted to examine the efficiency of hospitals. A study by [10] examined technical efficiency of the health centers in Ethiopia. Other study in Ethiopia is by [11] which used Stochastic Frontier Approach (SFA) to analysis the technical efficiency of 8 selected public hospitals. These few studies focused on the first stage of efficiency analysis. Therefore, this study seeks to analyze the technical efficiency and productivity of hospitals in eastern Ethiopia. This study also examines factors that determine the inefficiency of hospitals. The findings will deepen understanding of the extent of inefficiency and its causes in eastern Ethiopia. In the rest of the paper, methods and materials, result and discussions, and conclusion and recommendations are presented.

## Methods

This study was conducted on selected hospitals in eastern Ethiopia. The selected hospitals are from Eastern Hararghe (Oromia region), Harari region, Somali region, and Dire Dawa administration council. The included hospitals are both public and private. Panel data were collected from 12 hospitals for the period 2007/08 to 2012/13. The inputs include beds, health staff and drug supplies while the outputs include outpatient visits, inpatient days, and surgery.

### Method of data analysis

#### Data envelopment analysis (DEA)

Data envelopment analysis (DEA) involves the use of linear programming methods to construct a non-parametric piece-wise surface (or frontier) over the data [12]. DEA is based upon a comparative analysis of observed producers to their counterparts [13]. First, [14] coined DEA which had an input-oriented model with constant return to scale (CRS). Subsequently, variable returns to scale (VRS) model was also developed and introduced to the DEA literature by [15]. Furthermore, chance-constrained efficiency analysis was also integrated [16] to the DEA model.

##### CRS vs. VRS models

The assumption of CRS may not be feasible due to the presence of imperfect competition, government regulations, and constraints on finance that force firms to run at suboptimal scale [12]. For this reason, [15] developed a variable return to scale (VRS) which enables to capture the magnitude of scale effect. Linear programming model of VRS is like the CRS with some modification.

The mathematical relationship between VRS and CRS efficiency measurements is given by ${TE}_{CRS}={TE}_{VRS}(SE)$ [12]. “SE” denotes scale efficiency. This means that CRS technical efficiency of a firm can be decomposed into pure technical efficiency and scale efficiency (SE).

##### Malmquist productivity index

It is the measure of the relationship between the outputs of a hospital and the inputs used to produce those outputs. Productivity increase is manifested by rise in output per health worker hour and/or the use of more and/ or better health technology. In general, a productivity index is defined as the ratio of an output quantity index to an input quantity index, i.e. ${P}_t=\frac{Y_t}{X_t}$; Where: Pt is a productivity index; time t = 0…, T; Y_t_ is an output quantity index and X_t_ is an input quantity index. Each index represents accumulated growth from period 0 to period t.

The DEA-based Malmquist Productivity Index (MPI) is often opted to study efficiency and productivity changes over a given period. The model is preferred for several reasons: it does not require information on the prices of inputs and outputs rather on quantities of inputs and outputs; imposition of functional form of production technology is not required; it easily accommodates multiple hospital inputs and outputs; and it can be broken down into the constituent sources of productivity change - i.e. efficiency changes and technological changes [17].

Malmquist-DEA is applied to panel data to calculate indices of changes in Total Factor Productivity (TFP), technology, technical efficiency, and scale efficiency. The Malmquist Productivity Index (MPI) takes a value of more than one for productivity growth, a value of one for stagnation and a value of less than one for productivity decline. The output-oriented MPI is defined as the geometric mean of two periods’ productivity indices, subsequently broken down into various sources of productivity change [18].

#### Specification of the DEA model

The DEA model adopted is based on [19–21]; and many other model specifications that are applied in the health sector.

#### The constant returns to scale (CRS) model

The efficiency score of decision-making units that employs multiple input and output is defined as:

1 $$ Effeciency=\frac{weighted\  sum\  of\  hospital\  outputs}{weighted\  sum\  of\  hospital\  inpunts} $$

Following [14], if there are n hospitals, each with m hospital inputs and s hospital outputs, the relative efficiency score of a given hospital P is obtained by solving the following model:

$$ Efficienc{y}_P= Max\frac{{\displaystyle {\sum}_{r=1}^s}{U}_r{Y}_{r p}}{{\displaystyle {\sum}_{i=1}^m}{V}_i{X}_{i p}} $$

$$ s. t:\ \frac{{\displaystyle {\sum}_{r=1}^s}{U}_r{Y}_{r j}}{{\displaystyle {\sum}_{r=1}^m}{V}_i{X}_{i j}}\le 1;\  j=1;2;\dots; n $$

2 $$ {U}_r,\ {V}_i>0;{\forall}_r,{\forall}_i; r=1;2;.. s; i=1;2;.. m $$

Where:

${x}_{ij}$ = the amount of health system input i utilized by the j^th^ hospital;

${y}_{rj}$ = the amount of health system output r produced by the j^th^ hospital;

${u}_r$ = weight given to health system output r;

${v}_i$ = weight given to input i.

The functional programming model of equation (2) can be converted to a linear programming model by introducing the following constraint:

$$ {\displaystyle {\sum}_{i=1}^m{V}_i{X}_{i p}=1} $$

Thus, the relative efficiency score of hospital p can be obtained by solving the following equation:

$$ Max\  Efficienc{y}_P= Ma{x}_{u_r{v}_i}{\displaystyle {\sum}_{r=1}^s{U}_r{Y}_{r p}} $$

$$ S. t:{\displaystyle {\sum}_{r=1}^s{U}_r{Y}_{r j}}-{\displaystyle {\sum}_{i=1}^m{V}_i{X}_{i j}\le 0;{\forall}_i} $$

$$ {\displaystyle \sum }{V}_i{X}_{i p}=1 $$

3 $$ {U}_{r, }{V}_i>0; {\forall}_r,{\forall}_i $$

The first constraint implies that all hospitals are on or below the frontiers while the second constraint implies that the weighted sum of inputs for the hospital equals one.

#### The variable returns to scale (VRS) model

To separate the technical and scale efficiency scores, variable returns to scale (VRS) model is considered. In variable returns to scale, the data are enveloped more closely than the CRS model. The main advantage of the VRS model is that it enables an inefficient firm to be relatively compared to efficient hospitals of the same size only. Therefore, the relative efficiency score of hospital p can be obtained by solving the following equation:

$$ Max\  Effecienc{y}_P= Ma{x}_{u_r{v}_i}{\displaystyle {\sum}_{r=1}^s{U}_r{Y}_{r p}+{U}_0} $$

$$ S. t:{\displaystyle {\sum}_{r=1}^s{U}_r{Y}_{r j}}-{\displaystyle {\sum}_{i=1}^m{V}_i{X}_{i j}+{U}_0\le 0;{\forall}_i} $$

$$ {\displaystyle \sum }{V}_i{X}_{i p}=1 $$

4 $$ {U}_{r, }{V}_i>0; {\forall}_r,{\forall}_i $$

Where:

${U}_0$ = is the convexity constraint and its sign determines the returns to scale. If ${U}_0$ < 0 it indicates increasing returns to scale, if ${U}_0$ > 0 it is decreasing returns to scale and if ${U}_0$ = 0 it is constant returns to scale. The other notations are as given in the case of CRS model.

#### DEA like Malmquist model

The DEA like Malmquist model is used to obtain the DEA efficiency scores of all the sample periods observations. The model applies for panel data and calculates indices of total factor productivity (TFP) change, technological change, technical efficiency change and scale efficiency change. The output based Malmquist productivity change index of [18] is specified as follows:

5 $$ {M}_0\left({y}^{t+1},{x}^{t+1},{y}^t,{x}^t\right)={\left[\frac{D_0^t\left({x}^{t+1},{y}^{t+1}\right)}{D_0^t\left({x}^t,{y}^t\right)} X\frac{D_0^{t+1}\left({x}^{t+1},{y}^{t+1}\right)}{D_0^{t+1}\left({x}^t,{y}^t\right)}\right]}^{\frac{1}{2}} $$

Where:

${M}_0$ = measures productivity of the production point $\left({x}^{t+1},{y}^{t+1}\right)$ relative to the production point $\left({x}^t,{y}^t\right)$;

${D}_0^t\left({x}^{t+1},{y}^{t+1}\right)$ = represents the distance from the period t + 1 observation to the period t technology; and

${D}_0^t\left({x}^t,{y}^t\right)$ = represents the distance from period t observation to the period t + 1 technology.

If the value of ${M}_0$ is greater than one, it shows the existence of positive total factor productivity from period t to period t + 1 while a value less than one indicates a decline in total factor productivity. Further decomposition of equation (5) provides measures of efficiency change and technical change separately.

6 $$ {M}_0\left({y}^{t+1},{x}^{t+1},{y}^t,{x}^t\right)=\frac{D_0^t\left({x}^{t+1},{y}^{t+1}\right)}{D_0^t\left({x}^t,{y}^t\right)} x{\left[\frac{D_0^t\left({x}^{t+1},{y}^{t+1}\right)}{D_0^t\left({x}^t,{y}^t\right)} X\frac{D_0^{t+1}\left({x}^{t+1},{y}^{t+1}\right)}{D_0^{t+1}\left({x}^t,{y}^t\right)}\right]}^{\frac{1}{2}} $$

The first term measures efficiency change while the second term measures technical change in the two periods. An improvement of efficiency occurs from period t to period t + 1 if the ratio is greater than 1 (one). The output-oriented DEA model was estimated for CRS DEA and VRS DEA models.

#### Selection of input and output variables

The ultimate measure of output is an improvement in the quantity and quality of life. However, practical difficulties limit the use of outcomes approach [22]. Hence, hospital output is measured as an array of intermediate output (health services) that improves health status [23]. In this study, we included three output and three inputs based on the literature on health sector technical efficiency [4, 9, 24]. The outputs include outpatient department visit, inpatient days, and number of surgery. The inputs include total health staff, cost of drug supply, and capital input proxied by total beds.

#### Specification of the regression model

The DEA efficiency scores will be analyzed by regressing them against some characteristics of the hospitals to examine how these factors affect the (in) efficiency of hospitals. The censored Tobit model was used since the dependent variable is censored at zero from below. Like the studies of [19] and [25], in this study also the DEA scores are transformed into inefficiency scores using the following formula:

7 $$ Inefficiency\  score = \left(\frac{1}{DEA\  score}\right)-1 $$

The model is specified in the following form:

$$ {y}_i^{*}={\beta}_i{X}_i + {u}_i; $$

$$ {y}_i = {y}_i^{*}\  if\ {y}_i^{*} > 0;\  and $$

$$ {y}_i = 0\  if\ {y}_i^{*}\le 0 $$

Where:

*u*_*i*_ ~ *N* (0, *δ* ^2^);

${y}_i$ = the observed inefficiency score;

${\beta}_i$ = a $K x$ l vector of unknown parameters; and

${X}_i$ = a $K x1$ vector of explanatory variables.

Therefore, the empirical regression model is specified as:

8 $$ INEFF = {\alpha}_0+{\beta}_1 L\  size + {\beta}_2 B O R + {\beta}_3 Teacstat + {\beta}_4 dcstaf + {\beta}_5 opinpdays+{\beta}_6 impdoc + {\varepsilon}_i $$

The variables in the model are defined as follows:

**INEFF**: inefficiency scores.

***Size*** *:* The natural logarithm of numbers of bed is taken as a proxy to measure hospitals’ size.

***BOR***: It is the ratio number of inpatient days multiplied by 100 and divided by the available hospital beds multiplied by number of days in a year.

***Teachstat*** *:* It is teaching status dummy variable. It is 1 if it is teaching hospital and 0 other wise.

***Docstaff*** *:* This variable is measured by dividing the total number of medical doctors by the total staff of the hospital.

***Opinpdays*** *:* It is the outpatient visits as a proportion of inpatient days.

***Impdoc*** *:* It is the proportion of inpatients per medical doctor.

*α*_*0*_, β_1_, β_2_, β_3_, β_4_, β_5_, and β_6,_ are coefficients to be estimated and *ε*_*i*_ is the random disturbance term.

These variables used in the second stage (that is econometric analysis). We converted the efficiency results into inefficiency score using equation eight (8). Thus, we used this inefficiency score as dependent variable and run it against the above defined independent variables. Some of the determinants of the hospital facility technical efficiency include average length of stay, outpatient visit as proportion of the inpatient days, bed occupancy rate, doctors to staff ratio, teaching status of the hospital, proportion of inpatients per medical doctor, and bed size [9, 26, 27]).

### The data

Regional bureaus of health collect data on inputs and outputs and other health related data. The study was conducted based on the data obtained from these bureaus of health of each respective region (Oromia, Harari, Dire Dawa Administrative City Council, and Somali) in Eastern Ethiopia. The study used panel data of six year-round starting from 2007/08 to 2012/13 for each hospital. Having this panel data enables us analysis hospitals productivity changes overtime and allows us to control for unobserved heterogeneous characteristics of hospitals.

## Result and discussions

### Results of the study

#### Basic characteristics of hospitals

All (that is 12) hospitals in the Eastern Ethiopia are included in the study, except one for which the data was incomplete. The data collected covers the time between 2007/08 and 2012/13. Table 1 summarizes the basic characteristics of all hospitals and their respective average yearly inputs for the study period. Among the hospitals, eight were publicly owned while four were privately owned. In Harari region, 5 hospitals were selected for the analysis. Among the hospitals, Hiwot Fana, Jugal, Police and Army were public hospitals while Yimaj hospital was privately owned. From Dire Dawa, five hospitals were included: Dilchora and France hospital were public hospitals whereas Bilal, Yemariamwork and ART hospitals were privately owned. The other public hospitals considered in the analysis were Bisidimo and Karamara hospitals from East Hararghe zone of Oromia and Somali regions respectively. Regarding their year of establishment, France and Hiwot Fana hospitals were among the oldest hospitals where as ART and Yemariamwork were the youngest.

**Table 1:** Basic characteristics of hospitals and average yearly inputs 2007/2008–2012/2013

Name of hospital	Establishment	Staff			Average			
		Health	Administrative	Total	Salary	Drug	Recurrent	Bed
France	1911	14	23	37	591,637	381,532	414,248	68
Hiwot Fana	1941	225	196	421	6,406,047	1,765,662	6,278,350	204
Jegol	1951	151	112	263	3,495,719	831,116	3,899,048	118
Dilchora	1951	184	77	262	7,646,767	2,600,874	8,027,770	85
Karamara	1956	214	140	354	7,608,026	1,401,988	2,045,428	193
Bisidimo	1958	56	137	194	280,200	1,100,000	317,410	116
Police	1965	69	72	141	2,222,641	484,050	1,083,953	82
Army	1975	73	77	150	2,519,660	593,810	1,327,650	97
Yemariamwork*	2007	15	12	28	550,832	580,028	651,801	29
ART*	2007	20	5	25	1,914,318	560,297	1,168,419	28
Yimaj*	2006	45	10	55	982,006	113,572	3,877,606	54
Bilal*	2000	43	50	93	1,576,075	3,470,000	2,840,759	40

Source: own computation

Taking the average for the study period, Hiwot Fana hospital had the highest total staff which is 421 (196 administrative and 225 health staff) followed by Karamara, whereas ART had the lowest total staffs (5 administrative and 20 health staff) followed by Yemariamwork hospital.

Regarding the average yearly expenditure on salary, Dilchora hospital incurred the highest followed by Karamara hospital. The lowest expenditure on salary was incurred by Bisidimo. With respect to expenditure on drug, Bilal incurred the highest while Yimaj incurred the lowest. On the other hand, the average for yearly recurrent expenditure of the hospitals revealed that Dilchora hospital incurred the highest followed by Hiwot Fana. The lowest recorded was for Bisidimo followed by France.

The average number of bed for the study period revealed that Hiwot Fana had the highest number of bed followed by Karamara and Jugal. The lowest recorded was for ART followed by Yemariamwork hospital.

In this study, three major outputs of hospitals were considered for technical efficiency evaluation of hospitals. These three outputs were total yearly outpatient visit, total yearly inpatient days and total surgery performed in the respective hospitals. From Table 2, we can see that Dilchora had the highest average total outpatient visit followed by Hiwot Fana and Karamara hospitals. The lowest was recorded by Yemariamwork followed by ART and Yimaj.

**Table 2:** Hospitals average outputs 2007/2008–2012/2013

Name	France	Hiwotfana	Jegol	Dilchora	Karamara	Bisidimo	Police	Army	Yemariamwork	ART	Yimaj	Bilal
Outpatient visits	8740	35,424	26,191	64,272	28,484	7488	14,579	12,518	5602	5797	6832	12,256
Inpatient days	6308	26,520	13,789	44,233	24,122	3914	7207	5354	1979	1384	7914	8838
Surgery	193	949	1356	3825	1008	31	149	184	102	72	443	393

Source: various reports of hospitals and own computation

When we consider the total average yearly inpatient days, Dilchora had the highest average yearly inpatient days followed by Hiwot Fana and Karamara. The lowest was for ART followed by Yemariamwork and Bisidimo. About surgery, Dilchora had the highest total average yearly surgery followed by Jugal and Karamara. On the other hand, the lowest was recorded for ART followed by Yemariamwork and Police.

Table 3 shows that in the sampling period (2007/08to 2012/13), the average yearly output of the hospitals were 19,015 outpatient visits, 12,630 inpatient days and 750 number of surgeries, whereas the average yearly input of the total health staff, number of beds and expenditure on drugs was 92, 93, and birr 1,156,910, respectively.

**Table 3:** Means of outputs and inputs of the hospitals based on their ownership

No		Variable	Mean			Maximum		Minimum	
			All	Public	Private	Public	Private	Public	Private
I. Outputs	1	Outpatient visit	19,016	24,712	7622	64,273	12,256	7488	5602
	2	Surgery	751	1000	253	3825	443	149	73
	3	Inpatient days	12,631	16,431	5029	44,234	8839	3914	1384
II. Inputs	1	Health Staff	93	124	31	225	45	14	16
	2	Capital Input (Beds)	93	121	38	204	55	68	28
	3	Drug Supplies	1,156,911	1,144,879	1,180,974	2,600,874	3,470,000	381,532	113,572

Source: own computation

Overall, the public hospitals on average accounted for about 24,712, 999 and 16,431 outpatient visit, number of surgeries and inpatient days, respectively. Regarding inputs, on average over the sample period public hospitals accounted for about 123, 120 and 1,144,879 numbers of health staff, bed, and amount of birr of drug expenditure, respectively. From the above data, we can see that the share of the public in terms of average outputs and inputs is higher than the private hospitals except in drug expenditure.

#### Technical and scale efficiency

To separate the technical and scale efficiency in the health service production process which is often nonlinear, it is appropriate to assume output oriented variable returns to scale BCC model. Hence for this study we estimate the efficiency of hospitals assuming the variable returns to scale BCC model [21].

Tables 4 and 5 presents individual hospital’s technical and scale efficiency for the year 2007/08 to 2012/13. It reveals that in year 2007/08 and 2008/09 out of 12 hospitals 3 (25%) registered a constant return to scale technical efficiency (CRSTE) score of 100%. Therefore, 9 (75%) of hospitals in both 2007/08 and 2008/09 period has been run inefficiently given the assumption of constant return to scale.

**Table 4:** Hospital’s technical and scale efficiency for 2007/08 to 2009/10

	2007/08				2008/09				2009/10			
Hospitals	CRSTE	VRSTE	Scale	RTS	CRSTE	VRSTE	Scale	RTS	CRSTE	VRSTE	Scale	RTS
France	0.52	0.72	0.72	DRS	0.71	0.86	0.83	DRS	0.57	0.81	0.70	DRS
Hiwot Fana	0.54	0.55	0.99	DRS	0.55	0.59	0.93	DRS	0.86	0.88	0.98	DRS
Jegol	0.85	0.90	0.94	DRS	1.00	1.00	1.00	CRS	1.00	1.00	1.00	CRS
Dilchora	0.76	0.77	0.99	DRS	0.75	0.78	0.96	DRS	0.97	1.00	0.97	DRS
Karamara	0.98	1.00	0.98	IRS	0.39	1.00	0.39	IRS	0.58	1.00	0.58	IRS
Bisidimo	1.00	1.00	1.00	CRS	0.78	1.00	0.78	DRS	1.00	1.00	1.00	CRS
Police	0.85	1.00	0.85	IRS	0.83	1.00	0.83	IRS	0.70	1.00	0.70	IRS
Army	1.00	1.00	1.00	CRS	0.89	1.00	0.89	DRS	1.00	1.00	1.00	CRS
Yemariamwork	0.26	0.31	0.84	DRS	1.00	1.00	1.00	CRS	1.00	1.00	1.00	CRS
ART	0.62	0.70	0.88	IRS	0.19	0.24	0.77	DRS	0.47	0.52	0.92	DRS
Yimaj	0.96	1.00	0.96	DRS	0.73	0.82	0.89	IRS	1.00	1.00	1.00	CRS
Bilal	1.00	1.00	1.00		1.00	1.00	1.00	CRS	0.83	1.00	0.83	DRS
Mean	0.78	0.83	0.93		0.73	0.86	0.86		0.83	0.93	0.89	
SD	0.24	0.22	0.09		0.25	0.23	0.17		0.20	0.15	0.15	
Min	0.26	0.31	0.72		0.19	0.24	0.39		0.47	0.52	0.58	
Max	1.00	1.00	1.00		1.00	1.00	1.00		1.00	1.00	1.00	

Source: Own computation

**Table 5:** Hospital’s technical and scale efficiency in 2010/11 to 2012/13

	Hospitals efficiency 2010/11				Hospitals efficiency 2011/12				Hospitals efficiency 2012/13			
Hospitals	CRSTE	VRSTE	Scale	RTS	CRSTE	VRSTE	Scale	RTS	CRSTE	VRSTE	Scale	RTS
France	0.71	0.71	0.99	DRS	0.72	1.00	0.72	DRS	0.79	1.00	0.79	DRS
Hiwot Fana	0.71	0.71	0.99	DRS	0.53	0.54	0.99	DRS	0.56	0.62	0.90	DRS
Jegol	1.00	1.00	1.00	CRS	1.00	1.00	1.00	CRS	1.00	1.00	1.00	CRS
Dilchora	1.00	1.00	1.00	CRS	0.80	0.80	0.99	DRS	0.67	0.69	0.97	DRS
Karamara	0.52	1.00	0.52	IRS	0.64	1.00	0.64	IRS	0.66	1.00	0.66	IRS
Bisidimo	0.75	1.00	0.75	DRS	0.86	1.00	0.86	DRS	0.82	1.00	0.82	DRS
Police	1.00	1.00	1.00	CRS	1.00	1.00	1.00	CRS	1.00	1.00	1.00	CRS
Army	0.85	1.00	0.85	DRS	1.00	1.00	1.00	CRS	0.75	1.00	0.75	DRS
Yemariamwork	1.00	1.00	1.00	CRS	1.00	1.00	1.00	CRS	1.00	1.00	1.00	CRS
ART	0.52	0.54	0.97	DRS	0.58	0.64	0.91	DRS	0.50	0.65	0.77	DRS
Yimaj	1.00	1.00	1.00	CRS	1.00	1.00	1.00	CRS	1.00	1.00	1.00	CRS
Bilal	0.88	1.00	0.88	DRS	0.54	0.67	0.81	DRS	0.73	1.00	0.73	DRS
Mean	0.83	0.91	0.91		0.80	0.89	0.91		0.79	0.91	0.87	
SD	0.18	0.16	0.15		0.20	0.18	0.13		0.18	0.16	0.13	
Min	0.52	0.54	0.52		0.53	0.54	0.64		0.50	0.62	0.66	
Max	1.00	1.00	1.00		1.00	1.00	1.00		1.00	1.00	1.00	

Source: Own computation

On the other hand, 6 (50%) hospitals in the year 2007/08 and 7 (58.33%) of hospitals in the year 2008/09 registered a variable return to scale technical efficiency (VRSTE) score of 100%. Moreover, out of 12 hospitals, 3 hospitals (25%) in the year 2007/08 and 3 hospitals (25%) in the year 2008/09 were scale efficient. Regarding returns to scale, 3 (25%) of hospitals in the year 2007/08 and 3 (25%) of hospitals in the year 2008/09 manifested increasing returns to scale, respectively. Moreover, 6 (50%) and 6 (50%) of hospitals manifested decreasing returns to scale in the respective periods.

Individual hospitals’ technical and scale efficiency for the year 2009/10 and 2010/11 indicated that out of 12 hospitals, 5 (41.67%) registered a constant return to scale technical efficiency (CRSTE) score of 100%, whereas in the year 2010/11 again 5 (41.67%) hospitals registered a constant return to scale technical efficiency (CRSTE) score of 100%. Therefore, 7 (58.33%) of hospitals in the year 2009/10 and 7 (58.33%) of hospitals in the year 2010/11 were inefficient given the assumption of constant return to scale.

On the other hand, 9 (75%) of hospitals in both periods registered a VRS technical efficiency score of 100%. Moreover, 5 hospitals (41.67%) were scale efficient in both periods. Regarding returns to scale, 2 (16.67%) of hospitals and 1 (8.33%) of hospitals manifested increasing returns to scale in the respective periods. However, 5 (41.67%) of hospitals and 6 (50%) of hospitals manifested decreasing returns to scale in the respective periods.

Individual hospitals’ technical and scale efficiency for the year 2011/12 and 2012/13 showed that out of 12 hospitals, 5 (41.67%) of the hospitals in 2011/12 and 4 (33.33%) in 2012/13 registered a constant return to scale technical efficiency (CRSTE) score of 100%, respectively. Therefore, given the assumption of constant return to scale in the year 2011/12 7 (58.33%), and in the year 2012/13, 8 (66.67%) of hospitals run inefficiently.

On the other hand, 8 (66.7%) and 9 (75%) of hospitals in the respective periods registered a variable return to scale technical efficiency (VRSTE) score of 100%. Moreover, 5 (42%) and 4 (33%) of hospitals were scale efficient in respective periods. Regarding returns to scale, 1 (8.3%) of the hospitals in both periods manifested increasing returns to scale whereas 6 (50%) of the hospitals in 2011/12 and 7 (58%) in 2012/13 manifested decreasing returns to scale.

The average scale efficiency score was 93%, 86%, 89%, 91%, 91%, 87% in the respective years between 2007/08 and 2012/13. The average VRSTE scores of hospitals in Eastern Ethiopia stood at 91, 89, 91, 83, 86 and 93% respectively.

#### The required change in input and output to make inefficient hospitals efficient

Table 6 shows the required change in input reduction or output increase to make inefficient hospitals efficient for the study period. For example, in the year 2012/13, if inefficient hospitals were concerned with the output side, the inefficient hospitals would have increased outpatient visit by 3554, inpatient days by 1837 and surgery by 770 to become efficient. If hospitals were concerned with the level of inputs, the inefficient hospitals should have decreased their bed by 93 to be efficient. Specifically, Bisidimo hospital should increase outpatient by 3554 while army and police hospitals should also increase inpatient days by 511 and 1326, respectively. Moreover, army and police hospitals should also have increased the number of surgery operated by 365 and 404, respectively in 2012/13. This is if hospitals are concerned with output side. If the hospitals are concerned with level of input, Army, Police, and Bisidimo Hospitals should have reduced their bed size by 29, 7 and 57, respectively to be efficient.

**Table 6:** The Required Change in input and output to Make Inefficient Hospitals Efficient

	Year		Output			Input		
		Values	Outpatient visit	Impatient days	Surgery	Bed	Drug expenditure	Health staff
Required Change in input and output for all Hospitals for study period	2007/08	Total	8424	26,291	2711	77	1,007,946	70
		Mean	702	2191	226	6	83,995	6
	2008/09	Total	11,310	15,599	5186	72	1,872,247	16
		Mean	942	1300	432	6	156,021	1
	2009/10	Total	0	8659	38	130	626,184	57
		Mean	0	722	3	11	52,182	5
	2010/11	Total	3656	3256	0	118	0	15
		Mean	305	271	0	10	0	1
	2011/12	Total	6979	8142	877	170	689,659	14
		Mean	582	678	73	14	57,472	1
	2012/13	Total	3554	1837	770	95	0	0
		Mean	296	153	64	8	0	0
Total			33,923	63,783	9581	662	4,196,035	172
Mean			471	886	133	9	58,278	2

Source: Own computation

#### Malmquist Total Factor Productivity (MITFP) change

The study analyzed the differences in productivity over time based on Malmquist Total Factor Productivity (MTFP) index taking the year 2007/08 as the technology reference (see Table 7). Table 7 presents the Malmquist index summary of annual geometric means. In the last row (last column), we observe that on average MTFP decreased slightly by 3.6% over period 2007/08-2012/13. On average, the deterioration in MTFP was due to technical change rather than efficiency change. Hospital efficiency was increased by 1.2%, technical change decreased by 4.7%. The efficiency change was attributed to an increase in pure efficiency of 2.4% and a decline in scale efficiency of 1.2%.

**Table 7:** Malmquist index summary of geometric annual means (Output oriented)

Year	Efficiency change [A = (C*D)]	TE change [B]	PE change [C]	SE change [D = (A/C)]	MITFP change [E = A*B]
2008/09	0.923	1.01	1.036	0.891	0.932
2009/10	1.23	0.791	1.123	1.096	0.973
2010/11	1.01	0.898	0.99	1.02	0.907
2011/12	0.938	1.044	0.949	0.988	0.979
2012/13	0.988	1.047	1.031	0.958	1.034
Mean	1.012	0.953	1.024	0.988	0.964

Source: Own computation

MTFP change was 1.034in 2012/13. This shows that hospital productivity grew by 3.4% in 2012/13 compared to the reference year 2007/08. MTFP change was the highest in 2012/13 (MTFP = 1.034) and the lowest was in 2010/11 (0.907).

Table 8 provides a summary of the annual geometric mean values of the Malmquist Productivity Index (MPI) and its components for each hospital. Five (42%) out of 12 hospitals had MPI score greater than one, indicating growth in productivity. The hospitals include Karamara, Jugal, Bilal, France and Yemariamwork and their respective score is 0.6, 0.6, 7.7, 1.9 and 21.1%. The productivity growth in France was attributed to technical change only. Meanwhile, the productivity growth in Karamara, Jugal and Bilal was due to improvements in efficiency only. However, productivity change in Yemariamwork is due to both efficiency and technical change.

**Table 8:** Malmquist Index Summary of firm means

Year	Efficiency change [A = (C*D)]	TE change [B]	PE change [C]	SE change [D = (A/C)]	MITFP change [E = A*B]
Karamara	1.06	0.95	1.07	1.00	1.01
Army	1.01	0.96	1.03	0.98	0.97
Jugal	1.03	0.97	1.02	1.01	1.01
Police	0.98	0.98	0.98	1.00	0.95
ART	0.93	0.90	1.00	0.93	0.84
France	1.00	1.02	1.00	1.00	1.02
Yemariyam work	1.03	1.17	1.00	1.03	1.21
Yimaj	1.00	0.97	1.00	1.00	0.97
Dilchora	1.00	0.79	1.00	1.00	0.79
Bisidimo	1.12	0.85	1.14	0.99	0.95
Bilal	1.10	0.98	1.07	1.03	1.08
Hiwot Fana	0.91	0.95	1.00	0.91	0.86
Mean	1.01	0.95	1.02	0.99	0.96

Source: Own computation

On the other hand, 7 (58%) out of the 12 hospitals had Malmquist index score of less than one, indicating deterioration in productivity. Overtime productivity regression in Yimaj, Dilchora, Army and Bisidimo hospitals was due to deterioration in technical progress. However, productivity regression in the case of Police, ART and Hiwot Fana hospitals was due to decline in efficiency and technical progress.

##### Pure efficiency change

In Table 8, it is showed that 5 hospitals had an average pure efficiency change (PECH) score of greater than one. Hospitals registering a pure technical efficiency increase included Karamara (6.7%), Army (2.5%), Jugal (2.2%), Bisidimo (13.5%), and Bilal (7.4%). On the other hand, ART, France, Yemariamwork, Yimaj, Dilchora and Hiwot Fana hospitals registered a PECH score of one, indicating no change in efficiency at those hospitals between 2007/08 and 2012/13. However, Police hospital experienced a decline in PECH by 2.2%. The average PECH score for the entire sample was 1.024 during the study period, implying that PECH reduced efficiency by 4.2%.

##### Scale efficiency change

Scale efficiency change (SECH) is expressed as a value less than, equal to, or greater than one if a hospital scale of production contributes negatively, not at all, or positively, respectively, to productivity change [28]. The scale of production in Jugal, Yemariamwork and Bilal contributed positively to TFP change by a factor of 1.2, 3.2 and 2.6%, respectively.

France, Yimaj and Dilchora hospitals had a scale efficiency index value of one (1) meaning that those hospitals’ scale of production did not contribute to MTFP change. On the other hand, the SECH score for 5 hospitals was less than one (1) indicating that the scale of production in Karamara, ART, Army, Police and Hiwot Fana hospitals contributed negatively to productivity change by 0.5, 7.3, 1.8, 0.3 and 9%, respectively. The average SECH score for the entire sample was 0.988 indicating that the scale of production on average reduced efficiency change by 1.2%.

##### Technical change

Ten hospitals (83%) registered technical change (TECH) of less than one indicating a decline in technical progress. The lack of technological progress in Karamara, Army, Jugal, Police, ART, Yimaj, Dilchora, Bisidimo, Bilal, and Hiwot Fana led to decrease in TFP (Total Factor Productivity) of 5.3, 3.8, 2.7, 2.3, 9.6, 2.8, 21.3, 2.2, 5.6 and 5.4%, respectively. France and Yemariamwork hospitals registered technical progress between the period t and t + 1 of 1.9 and 17.3%, respectively.

#### Tobit regression model results

In this study, we used random effect Tobit model. The panel data is for 6 periods running from 2007/08 to 2012/13. To analyze the determinants of inefficiency of hospitals, the technical efficiency score of hospitals was converted to inefficiency score of hospitals. Subsequently, the inefficiency score was used as a dependent variable and regressed against hypothesized determinants (Size, Teachstat, BOR, Dcstaf, Opvinpdays and impdoc) using a censored Tobit model. Table 9, indicates that among the explanatory variables included in the analysis, four of them (Teachstat, Dcstaff, Opinpdays, impdoc) were found statistically significant while the remaining two were insignificant.

**Table 9:** Summary of the Censored-Tobit regression analysis

Explanatory variables	Coefficient	Std. Err	Z
Lsize	−1.715701	2.451574	−0.7
Teachstat	3.034462**	1.340485	2.26
BOR	−0.0065286	0.0048885	−1.34
Dcstaf	−26.65755*	7.197905	−3.70
Opvinpdays	−0.3298481***	0.1885694	−1.75
Impdoc	−4.807009*	1.479342	−3.25
Constant	23.71757	6.968924	3.4
observations 72; 29 left-censored observations at Ineff ≥ 0; 43 uncensored observations			
0 right-censored observations chi2 (7) = 28.87 Prob > chi2 0.0002			

*p ≤ 0.01 ** *p* ≤ 0.05*** *p* ≤ 0.1

Teaching status (**Teachstat**) of the hospital is positively related with inefficiency score at 5% level of significance. This implies that being a teaching hospital reduces the expected efficiency score by 3.03.

The proportion of **Dcstaff** (medical doctors to the total staff) is negatively related with inefficiency and statistically significant at one (1) % level of significance. This implies that for a one unit increase in Dcstaff (medical doctors to the total staff ratio); there is a 3.75 unit decrease in inefficiency score.

The coefficient for Opinpdays (outpatient visits to inpatient days ratio) has a negative sign that is consistent with our a priori expectation and significant at 10% level of significance. A one unit increase in the ratio of outpatient visit to inpatient days would lead to a decrease in hospital expected inefficiency score by 1.75.

The coefficient of the **Impdoc** (proportion of inpatients treated per medical doctor) is negatively related to inefficiency score and statistically significant at one percent level of significance. It shows that the number of inpatients per medical doctor has negative relationship with inefficiency score. This implies that a one percent increase in the ratio of inpatient per doctor would drop the predicted value of inefficiency score by 3.25%.

### Discussions

#### Technical and scale efficiency of the hospitals

The average Variable Returns to Scale Technical Efficiency (VRSTE) scores of hospitals in Eastern Ethiopia were 91, 89, 91, 83, 86 and 93% during the period, respectively. This finding implies that if run efficiently the hospitals could have produced 9, 11, 9, 17, 14 and 7% more output (outpatient department visit, impatient days and number of surgery) for the same volume of inputs. The average VRSTE of hospitals in Eastern Ethiopia exhibit similarity with hospitals in Northern and Western Cape Provinces (82–82.8%) [29], Kwazulu Natal province of South Africa (90.6%) [30], and Kenya (84%) [31]. On the other hand, the average VRSTE scores were higher than those of Angola (65.8–67.5%) [8], Ghana (61%) [21], Zambia (67%) [32], Benin (63.3–85.8%) [9] and Namibia (62.7–74.3%) [33]. But it is lower than those for Uganda (90.2–97.3%) [34].

The average scale efficiency scores were 93%, 86%, 89%, 91%, 91%, 87% in the respective years between 2007/08 and 2012/13. These average scale efficiency scores were within the range of those for Angola (81–89%) [35], Kenya (90%) [31], and South Africa (Eastern, Northern and Western Cape Provinces) (82.5–90%) [29]. However, it is higher than those for Benin (41.9–73.6%) [6], Ghana (81%) [21], Namibia (73.2–83.7%) [33], and Zambia (80%) [32]. Moreover, the average scale efficiency scores for Botswana were lower than those of Kwazulu-Natal Province of South Africa (95.3%) [30], and Uganda (97.5%) [34].

#### The required change in input and output to make inefficient hospitals efficient

Table 6 shows the required change in input reduction or output increase to make inefficient hospitals efficient for the study period. In this vein, if inefficient hospitals were concerned with the output side, the inefficient hospitals would have increased outpatient visit, inpatient days and surgery to become efficient. If hospitals were concerned with the level of inputs, the inefficient hospitals should have decreased their bed to be efficient.

Accordingly, the finding suggest that for hospitals with outputs that fall short of DEA targets, health policymakers could improve efficiency by improving access to and utilization of underutilized maternal and neonatal health and other services that has been underutilized. This may call for a multi-pronged strategy involving: Utilizing health promotion strategies and techniques such as: social mobilization, advocacy; social marketing; information, education, and communication (IEC); regulation and legislation; partnerships and alliances with public, private, non-governmental organization and civil society: and inter-sectoral action to address determinants of health to improve the use of underutilized health services [36].

It is also possible to provide access to universal health services through pooled pre-paid contribution collected based on ability to pay through the tax based funding [37]. The other alternative, if it is not possible to solve the inefficiency problem through the improved utilization of hospital health services, is to transfer the excess health staff, beds and drugs to health clinics, health posts and other health stations located in remote areas. However, an efficiency analysis of these lower level health facilities should guide this.

#### Total factor productivity change

The average MTFP of 0.964 for hospitals in eastern Ethiopia was comparable to those obtained in China inland of 0.985 [38], Greece of 0.986–0.988 [39], Ireland country of 0.977 [40], South Africa of 0.879 [19], and Taiwan 0.788 [41]. Unlike hospitals in Eastern Ethiopia, several countries hospitals had an average MTFP score greater than one signifying productivity growth. Portugal had 1.042 [42] Ireland regional hospitals had 1.028 [43], India district hospital had 1.235 [28], Angola municipal hospital had 1.045 [35] and China coastal hospitals had 1.121 [38].

Technical progress registered by hospitals may have been the result of applying better techniques with regard to both physical and human capital which allowed greater output with health system inputs held constant. This improvement could also have resulted from increases in motivation and/or skill of the health workforce.

Technical progress (or regression) depends on different factors. These factors may include: the availability of appropriate health technology which require minimum skill with accompanying inputs and institutional changes. The existence of close cooperation of health policy makers and the hospital management is also essential. Moreover, it is also important to equip the relevant health workforce so that they will be able to use the new technology efficiently [44].

#### Determinants of inefficiency of hospitals

Random effect Tobit model showed that teaching status of the hospital (Teachstat), medical doctor total staff ratio (Dcstaff), outpatient visits to inpatient days ratio (Opinpdays), and proportion of inpatients treated per medical doctor (impdoc) significantly determine the hospitals inefficiency in the study area.

Accordingly, the fact that teaching status of the hospital (**Teachstat**) has hospital is positively effect on inefficiency score of the hospital implies that being a teaching hospital reduces the expected efficiency. This might be related to the focus that these teaching hospitals are given in terms of materials and other necessary inputs. These teaching hospitals offer specialized services that attract patients. The hospital that provides both health services and training are less efficient than other hospitals. This finding may explain that teaching hospitals are a place where knowledge, skills, and experience are obtained through practical training, learning, and demonstration in the hospital. Doing these learning and teaching alongside with provision of the health services may complicate and adds to inefficiency of the hospitals. This is may be because of teaching hospitals may not get focused on the activities as they care for both the academics and health services provision. Moreover, teaching hospitals care much the training, education, skills, that their staffs and students must acquire from the hospital.

The fact that the proportion of medical doctors to the total staff (**Dcstaff**) negatively related to inefficiency implies that for an increase in ratio of medical doctors to the total staff there is a decrease in inefficiency score of hospitals. In the hospital, medical doctors are the staffs that have the highest level of education and training, and this may positively increase the efficiencies of the hospital. It may also be related to the spillover effect of the doctors to the other staffs. Additionally, this might be related to the improvement that might occur in facilitating service delivery associated with increasing of doctors as their quantity is few in developing country compared to the number of patient each serve. In contrast to this result, a study conducted in West Bengal in India by [45] discovered that doctor staff ratio negatively affected the technical efficiency of hospitals.

The negative relation between outpatient visits to inpatient day ratio (Opinpdays) and hospital inefficiency score indicates that an increase in the ratio of outpatient visit to inpatient days would lead to a decrease in hospital expected inefficiency score of hospitals. This result also agrees with the result obtained by [4] in the study of determinants of technical efficiency of hospitals in Eritrea. Similarly, [19] also discovered the same result in the case of determinants of technical efficiency of hospitals in South Africa.

The proportion of inpatients treated per medical doctor (**Impdoc**) is negatively related to inefficiency score of hospitals. This shows that the number of inpatients per medical doctor has negative relationship with inefficiency score. This implies that an increase in the ratio of inpatient per doctor would drop the predicted value of inefficiency score of hospitals. This finding may explain that there is a need to efficiently use available doctors and hospitals that provide health services. By doing so, efficiency and standard ratio of inpatients to the doctors can be maintained.

## Conclusion and recommendation

This study attempts to analysis technical efficiency of hospitals in eastern Ethiopia. To achieve the objective of the study DEA, MTFP, and Tobit model are used. The DEA used to estimate the efficiency score of hospitals, MTFP used to analyses productivity change of hospitals and Tobit model used to analysis factors that determine inefficiency of hospitals.

Estimate of DEA indicates that under a VRS assumption, 6 (50%), 5 (42%), 3 (25%), 3 (25%), 4 (33%) and 3 (25%), while under a CRS assumption, 9 (75%), 9 (75%), 8 (67%), 7 (58%), 7 (58%) and 8 (67%) of the 12 hospitals were run inefficiently between 2007/08 and 2012/13. The results also indicate that 9 (75%), 9 (75%), 7 (58%), 7 (53%), 7 (58%) and 8 (67%) of the 12 hospitals were scale inefficient between 2007/08 and 2012/13. The estimate of the MTFP indicates that among the 12 hospitals, 7 (58%) experienced MTFP deterioration over the six years. The Tobit model indicates that teaching hospital is less efficient than other hospitals. The Tobit regression model further indicates that medical doctor to total staff ratio, the proportion of outpatient visit to inpatient days, and the proportion of inpatients treated per medical doctor were negatively related with technical inefficiency of hospitals.

Based on the findings, the following policy recommendations are forwarded. Further improvements can be made in the hospitals’ efficiency by taking the following policy measures.

- ➢ Hospitals should monitor their services delivery efficiency and need to identify inputs that are underutilized. This may help hospitals identify which input need to increase or decrease or transfer to other health services facilities so that the use of underutilized health sector services will be promoted.
- ➢ Hospitals should monitor their services delivery efficiency and need to identify output that fall short of targets. This may help hospitals identify which output/services need to increase or decrease so that they may deliver better services. In this regard, it is crucial to institutionalize efficiency monitoring of health facilities within health management information system. This can be implemented by either having section with in structure of each hospital that study the efficiency or regularly hiring study agent. By doing so hospitals may maintain their pure efficiency, scale efficiency and/or technical efficiency and thereby total factor productivity in delivering the service would increase.
- ➢ Policy interventions that increase utilization of underutilized hospital outpatient health services and reduce the average length of stay, increase doctor to other staff ratio and the number of inpatients treated per doctor would contribute to improve the technical efficiency of hospitals. This may be achieved by provision of the capacity building like training for the staff members of the hospitals on efficient resource utilization and service delivery. Increasing the health staffs that considers the population of the study area and standard of the health staff to patient ratio may help improve the efficiency of the hospitals. This can be achieved through hiring additional health staffs, and upgrading the capacity of the existing staffs through provision of on job training.

## Abbreviations

CRS: Constant return to scale
CRSTE: Constant return to scale technical efficiency
DEA: Data envelopment analysis
MITFP: Malmquist Total Factor Productivity
MPI: Malmquist Productivity Index
SFA: Stochastic Frontier Approach
TFP: Total factor productivity
VRS: Variable returns to scale
VRSTE: Variable return to scale technical efficiency
